# Evaluation of Corneal Biomechanical Changes After Collagen Crosslinking in Patients with Progressive Keratoconus by Ocular Response Analyzer

**DOI:** 10.4274/tjo.56750

**Published:** 2018-09-04

**Authors:** Raciha Beril Küçümen, Berna Şahan, Canan Aslı Yıldırım, Ferda Çiftçi

**Affiliations:** 1 Yeditepe University Faculty of Medicine, Department of Ophthalmology, İstanbul, Turkey; 2 Dokuz Eylül University Faculty of Medicine, Department of Ophthalmology, İzmir, Turkey

**Keywords:** Keratoconus, collagen crosslinking, corneal biomechanics

## Abstract

**Objectives:**

To evaluate corneal biomechanics before and after collagen crosslinking (CXL) in patients with progressive keratoconus.

**Materials and Methods:**

In this prospective study, CXL was performed under topical anesthesia after removal of the epithelium (epi-off technique) by applying ultraviolet A (UVA) light at a wavelength of 365 nm and power of 3 mW/cm2 or 5.4 joule/cm2. Isoosmolar 0.1% riboflavin solution was administered before and during UVA irradiation. In addition to ophthalmologic examination, ocular response analyzer measurements were performed pre- and postoperatively. Corneal hysteresis (CH), corneal resistance factor (CRF), corneal compensated intraocular pressure (IOPcc), Goldmann-correlated intraocular pressure (IOPg), and central corneal thickness (CCT) were recorded.

**Results:**

The study included 35 eyes of 30 patients with progressive keratoconus. The mean age was 28.2±6.5 years and postoperative follow-up time was 20.2±14.7 months (range: 6-74 months). The mean CH was 8.60±1.23 mmHg preoperatively, 8.96±2.05 mmHg in the early postoperative period (1-6 months), (p=0.28) and 8.96±1.28 mmHg in the late postoperative period (10-29 months) (p=0.48). Mean CRF was 7.13±1.50 mmHg preoperatively, 8.48±2.16 mmHg in the early postoperative period (p=0.009), and 7.71±1.29 mmHg in the late postoperative period (p=0.40). Mean IOPcc was 12.78±2.34 mmHg preoperatively, 15.38±4.21 mmHg in the early postoperative period (p=0.12) and 13.68±3.61 mmHg in the late postoperative period (p=0.48). Mean IOPg was 9.56±2.73 mmHg preoperatively, 13.01±4.45 mmHg in the early postoperative period (p=0.046), and 10.86±3.47 mmHg in the late postoperative period (p=0.44). Mean CCT was 484.43±41.26 µm preoperatively, 474.16±64.74 µm in the early postoperative period (p=0.70), and 470.38±33.64 µm in late postoperative period (p=0.71).

**Conclusion:**

CXL is a treatment modality believed to affect corneal biomechanics in keratoconus, but the results of larger patient series with longer follow-up periods may enable a better evaluation.

## Introduction

Keratoconus is a progressive, non-inflammatory degenerative disease in which the cornea gradually thins and becomes cone-shaped.^1,2^ Decreased corneal stability leads to stromal thinning and protrusion. It causes irregular corneal astigmatism and myopia, thus reducing visual acuity. Visual impairment often appears in adolescence. Even in cases of bilateral involvement, the eyes are affected asymmetrically.^3^ Although the prevalence of keratoconus varies depending on ethnic and geographical factors, it is reported as 50-600 per 100,000 in the general population.^4^

The course of keratoconus is highly variable and disease stage affects the treatment strategy. In the early stages, irregular astigmatism can be treated with hard or custom-made contact lenses.^5,6^ Intracorneal ring segment implantation is another treatment option for patients who are averse to or cannot tolerate wearing contact lenses, and it enables visual rehabilitation by correcting refraction.^6,7^ Intracorneal rings are also thought to contribute to corneal stability by affecting the biomechanics of the cornea.^8,9^ When these options are inadequate in patients with severe irregular astigmatism and stromal scarring, deep anterior lamellar keratoplasty or penetrating keratoplasty may be preferred.^4,10^

Collagen crosslinking (CXL) has gained attention in recent years as a treatment approach to keratoconus. It is universally accepted for the treatment of advanced keratoconus. CXL halts or delays the progression of the disease, thus reducing the need for lamellar or penetrating keratoplasty. In the corneal stroma, riboflavin (vitamin B2) and ultraviolet-A (UVA) undergo a photochemical reaction with environmental oxygen and generate free oxygen radicals. This photochemical reaction forms additional covalent bonds between the collagen fibrils in the stroma, thus reinforcing the structure of the corneal stroma. Therefore, CXL induces a process that influences and reshapes the biomechanics of the cornea.^11^

In this study, we aimed to use an ocular response analyzer (ORA, Ocular Response Analyzer, Reichert Ophthalmic Instruments, Corp., NY, USA) to examine eyes that underwent CXL treatment at our clinic due to progressive keratoconus, and evaluate the biomechanical changes that may occur in the cornea.

## Materials and Methods

Thirty-five eyes of 30 patients who were diagnosed with progressive keratoconus at the Yeditepe University Eye Center between September 2011 and August 2015 were included in this prospective study. CXL treatment was planned for all patients; the research protocol was explained to all patients before the intervention and informed consent forms were obtained. The study was conducted in compliance with the Declaration of Helsinki principles and was approved by the hospital ethics committee. The patients underwent preoperative and postoperative examination including ophthalmologic examination, corneal topography using two different technologies, the GALILEI™ Dual Scheimpflug Analyzer (Ziemer Group AG, Switzerland) and the Wavelight Allegro Topolyzer (Alcon Laboratories, Inc., Fort Worth, TX, USA), and ORA measurement. The patients were clinically and topographically diagnosed with keratoconus based on clinical and biomicroscopic findings such as a scissoring reflex on retinoscopy, Munson’s sign, thinning of the cornea, Vogt striae, and Fleischer rings, and keratoconus patterns and corneal index changes on corneal topography. An increase of more than 1.00 diopter (D) in the vertical keratometry value within the last 12 months and/or a 0.50 D increase in spherical refraction, and a 1.00 D increase in the cylindrical refraction value were accepted as criteria for progression.^11,12^

Study inclusion criteria were being 18-40 years of age, having no ocular pathology other than keratoconus, and having a minimum corneal thickness of 400 microns and progression of keratoconus in the last 12 months. Patients with herpetic keratitis, severe dry eye, blepharitis, corneal infection, corneal scarring, and/or a history of autoimmune disease and patients who had undergone ocular surgery were excluded from the study. None of the patients had a history of smoking or diabetes. Pregnant women and breastfeeding mothers were also not included in the study.

The ORA delivers a rapid air pulse to the cornea during measurement, similar to air-puff tonometers; it then makes calculations by eliminating the potential interference between the pressure and the force. The first recording is acquired as the air jet creates the first applanation in the cornea. As the air jet continues to exert pressure, the cornea becomes concave. The air pulse is discontinued within milliseconds (ms). The cornea flattens again with the decrease in force, and the second recording is made during the second applanation. The cornea then returns to its normal convex state. Measurements are taken from the 3.0 mm-diameter area of the central cornea. An electrooptic detector monitors the area for 20 ms. A graph is made from the recorded values (Graphic 1). The two peaks clearly visible at the top of the graph indicate the first and second applanations (P1 and P2). The viscoelastic nature of the cornea results in different pressure values at the applanations. The intersection points of the applied pressure and applanation pressure curves are identified, and the difference in height between them is defined as corneal hysteresis (CH) (Graphic 1). In other words, CH refers to the energy the cornea loses in order to return to its former state after being deformed by the airjet during ORA measurements. Another parameter provided by the ORA is corneal resistance factor (CRF). This parameter shows the total viscoelastic resistance of the cornea. The value is determined by calculating the linear function between the two applanation pressures (P1 and P2). The formula is defined as CRF=k1 x (P1-0.7xP2) + k2, where k1 and k2 are constants. Other ORA parameters are corneal compensated intraocular pressure (IOPcc), Goldmann-correlated intraocular pressure (IOPg), and central corneal thickness (CCT). The CCT measurement is taken after the ORA measurement with an ultrasonic pachymeter adapted to the device.

### Surgical Technique

CXL was performed under sterile conditions and surgical microscopy. After applying topical anesthesia (2-3 drops of 0.5% proparacaine hydrochloride) to the target eye, the periorbital area and lids were cleaned with 10% povidone iodine, as in preparation for refractive surgery. With a sterile drape, the eyelashes were drawn back, the eye was covered, and a blepharostat was placed. The ocular surface was irrigated with a balanced salt solution (BSS). Several measurements were taken from the cornea using an ultrasonic pachymeter (PacScan 300AP, Sonomed Inc., NY, USA) and the minimum corneal thickness of at least 400 µm was reconfirmed. The cornea was prepared by placing 20% alcohol in a 9 mm diameter ring centered on the cornea for 45 seconds. After rinsing the surface with ample BSS, the corneal epithelium was removed as a flap. A solution containing riboflavin (0.1% riboflavin, 20% dextran) was instilled every 3 minutes for the first half hour in accordance with the Dresden protocol.^16^ During the second half hour, 365-nm UVA at a dose of 3 mW/cm^2^ or 5.4 J/cm^2^ was applied to the corneal apex from a distance of 5 cm using a CXL device (PESCHKE Trade CCL-VARIO Cross-linking). During irradiation, the riboflavin solution was instilled every 2.5 minutes and artificial tears (3 mg/mL hydroxypropylmethylcellulose, Tears Naturale^®^ II, Alcon, Belgium) were instilled between drops of riboflavin in order to prevent dehydration of the cornea. Topical antibiotic (0.5% moxifloxacin, 4 times daily), topical corticosteroid (1% prednisolone acetate, 4 times daily), and artificial tear drops were prescribed postoperatively.

### Statistical Analysis

In addition to ophthalmologic examination, the patients were evaluated with ORA before surgery, in the early postoperative period (1-6 months), and in the late postoperative period (10-29 months). ORA measurements were made between 10:00 and 12:00 in the morning. At least 3 measurements were taken from the patients and the most qualitative measurement with the highest waveform score was used for evaluation. In the statistical analysis, the pre-CXL and post-CXL CH, CRF, IOPg, IOPcc, and CCT values were evaluated using a dependent paired-samples t-test in SPSS (Statistical Package for the Social Sciences Inc., Chicago, IL, USA) software. Change in a parameter with a p value of <0.05 was considered a significant difference.

## Results

Thirty-five eyes of 30 patients were included in the study. Twenty-two patients (73%) were male and 8 (27%) were female. The mean age was 28.2±6.5 years (18-38 years). The mean postoperative follow-up period was 20.2±4.7 months (10-29 months) (Table 1). The results of 3 male patients (4 eyes) and 1 female patient (1 eye) who were lost to follow-up after the first month were excluded from the statistical analysis.

Table 2 shows the uncorrected and best corrected visual acuity, refraction, and topographic results of the patients before and after surgery. Significant improvement was found in all parameters (p<0.05).

Preoperative and early and late postoperative ORA parameters are shown in Table 3.

The mean CH was found to be higher in the early and late postoperative periods compared to the preoperative value, but the difference was not statistically significant (p_1_=0.25, p_2_=0.48). The mean CFR was significantly higher in the early postoperative period compared to the preoperative value (p_1_=0.009), but there was no statistically significant increase in the late postoperative period compared to the preoperative value (p_2_=0.40). There was a significant increase in mean IOPg in the early postoperative period compared to the preoperative value (p_1_=0.46). The late postoperative mean IOPg was higher than the preoperative value but the difference was not statistically significant (p_2_=0.44). Mean IOPcc was higher in the early and late postoperative period than the preoperative value, but this difference was also not statistically significant (p_1_=0.12; p_2_=0.48). Early and late postoperative mean CCT was thinner in the early and late postoperative period compared to the preoperatively, but the difference was not statistically significant (p_1_=0.70; p_2_=0.71). There was no significant difference between the early and late postoperative period means of ORA parameters (CH, CRF, IOPg, IOPcc, CCT) (Table 3, p_3_ values).

## Discussion

In recent years, it has been reported that CXL therapy slows, stops, and even reverses keratoconus.^17,18,19^ In vitro studies have demonstrated that the treatment increases the number of crosslinks in the stroma and thus enhances the biomechanical resistance of the cornea.^20,21^ In a 2003 in vitro study conducted with a strip extensometer, Wollensak et al.^22^experimentally demonstrated that Young’s modulus, which indicates the biomechanical rigidity of the cornea, increased 4.5 times in the human cornea and 1.8 times in the porcine cornea after CXL. However, this method is not suitable for clinical use because it is done with stripped corneal tissue.

Devices that assess corneal biomechanics in vivo are the ORA, the Corvis tonometer (Corvis^®^ ST OCULUS Optikgeräte GmbH, Wetzlar, Germany), and applanation resonance technology.^23,24^ The ORA is most commonly used in the clinic for evaluating corneal biomechanics, and there are many studies based on ORA results after various ocular pathologies and eye surgeries.^9,13,14,15,25,26,27,28^ ORA studies performed in keratoconus have reported lower CH and CRF parameters compared with normal eyes.^16^ Following corneal transplantation in eyes with advanced keratoconus, CH and CRF were increased but were found to be lower compared to normal eyes.^10^

In our study, mean CH was increased in the early and late post-CXL periods compared to the preoperative level, but the difference was not statistically significant. On the other hand, mean CRF, which is considered an important parameter in keratoconus, has been found to be significantly increased in the early post-CXL period.^29^ Mean CRF also increased in the late post-CXL period compared to the preoperative period, but the change was not statistically significant.

The low IOP measurements in eyes with keratoconus are attributed to low corneal rigidity and corneal thinning.^30^ In our study, we observed increases in both mean IOPg and IOPcc in the early and late postoperative periods after CXL. However, only the increase in IOPg seen in the early postoperative period was statistically significant. Mean CCT showed statistically insignificant thinning in the early and late post-CXL periods. This thinning may be explained by the collagen fibers becoming more compact due to the increased crosslinkage in the stroma and scar formation.^12,30^

ORA studies performed in eyes with keratoconus following CXL report different results regarding biomechanical changes. Statistically insignificant increases in CH and CRF values were reported at 6 months after treatment in 2 studies and at 1 year after treatment in another.^31,32,33^ Çağıl et al.^12^ also found a nonsignificant increase in CH or CRF values at postoperative 1 and 6 months, but observed a significant decrease in CCT. Vinciguerra et al.^19^ reported significant increases in CH and CRF values at postoperative 1 month. However, they found no significant differences at postoperative 6 and 12 months compared to the preoperative values, and reported statistically insignificant reduction in CCT at postoperative 12 months. Greenstein et al.^34^ also found a significant increase in CRF in 1 and 3 months, but found no significant difference at 1 year. While our results are consistent with the literature, the small differences among these studies are also noteworthy.

CXL therapy aims to increase the rigidity and resistance of the cornea. An increase in the values of parameters that measure corneal biomechanics is expected after CXL. Therefore, theoretically, a statistically significant increase would be expected after CXL in the biomechanical indicators assessed by the ORA device, especially the CH and CRF values. In our study, we observed that two of the ORA parameters (CH and IOPg) increased significantly in the early post-CXL period, while four of them (CH, CRF, IOPg, IOPcc) increased in the late period, albeit statistically insignificantly. The fact that our results are largely statistically insignificant may be due to various reasons. The first reason is the low number of patients, which is the main limitation of our study. The second may be the collagen lamellae becoming compact after CXL. As the cornea becomes thinner, the measurements decrease. In the present study, mean CCT measurements showed reductions of 10 µm in the early postoperative period and 14 µm in the late postoperative period, which may have caused the parametric values to be lower than expected. Another reason may be that each eye with keratoconus has different configuration, pachymetric, topographic, and therefore biomechanical properties. It is argued that because the cornea is not homogeneous, the ORA device may not be technologically sufficient for measurements. Other studies have similarly addressed the possibility of optical irregularities in ectatic corneas obscuring actual biomechanical changes by altering ORA signals.^35,^^36^ With time, the development of more precise versions and/or new devices may yield more meaningful results.

## Conclusion

In summary, CXL is a promising new treatment modality for keratoconus patients and may affect corneal biomechanics. Larger patient series and more advanced technologies are needed to fully understand corneal biomechanics and to quantitatively and precisely assess them. Devices that can accurately measure these changes and are suitable for clinical use have not yet been developed, including the ORA. To understand the mechanism of action of this therapy on the cornea, there is a need for multicenter, randomized, prospective studies including large patient populations with long postoperative follow-up periods, which will provide more statistically valuable results.

## Figures and Tables

**Table 1:**
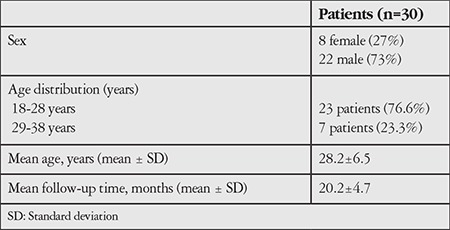
Demographic characteristics of the patients

**Table 2:**
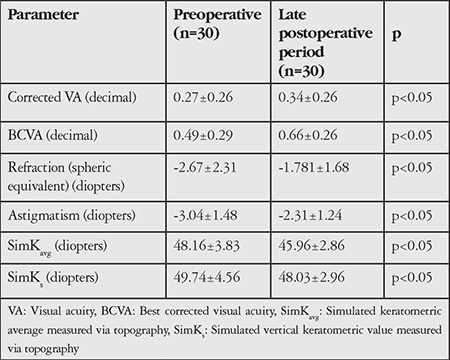
Patients’ preoperative and late postoperative visual acuity, refraction, and topographic findings

**Table 3:**
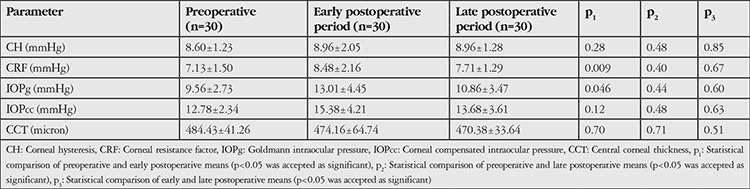
Preoperative, early postoperative, and late postoperative ocular response analyzer results of patients who underwent collagen crosslinking therapy

**Graphic 1:**
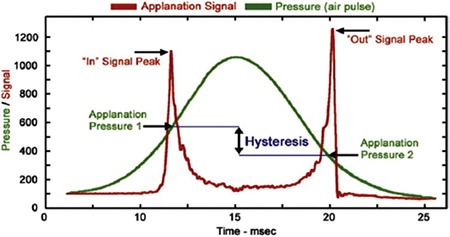
The two peaks on the red line represent the first and second applanations (P1 and P2). The green curve shows the air pressure applied by the device. The difference in height between the two intersection points corresponds to the histeresis value in mmHg (translated from the manufacturer’s user guide)
